# An overview of torrefied bioresource briquettes: quality-influencing parameters, enhancement through torrefaction and applications

**DOI:** 10.1186/s40643-022-00608-1

**Published:** 2022-11-28

**Authors:** M. A. Waheed, O. A. Akogun, C. C. Enweremadu

**Affiliations:** 1 Department of Mechanical Engineering, College of Science, Engineering and Technology, University of South Africa, Science Campus, Florida, 1709 South Africa; 2 Agricultural Mechanization and Sustainable Environment Programme, Centre of Excellence in Agricultural Development and Sustainable Environment, Federal University of Agriculture, P. M. B, Abeokuta, 2240 Nigeria

**Keywords:** Biomass energy value, Bioresource briquette qualities, Torrefaction pre-treatment, Briquette quality parameters

## Abstract

In recent years, the need for clean, viable and sustainable source of alternative fuel is on the rampage in the global space due to the challenges posed by human factors including fossil induced emissions, fuel shortage and its ever-rising prices. These challenges are the major reason to utilize alternative source of energy such as lignocellulosic biomass as domestic and industrial feedstock. However, biomass in their raw form is problematic for application, hence, a dire need for torrefaction pre-treatment is required. The torrefaction option could ameliorate biomass limitations such as low heating value, high volatile matter, low bulk density, hygroscopic and combustion behaviour, low energy density and its fibrous nature. The torrefied product in powder form could cause air pollution and make utilization, handling, transportation, and storage challenging, hence, densification into product of higher density briquettes. This paper therefore provides an overview on the performance of torrefied briquettes from agricultural wastes. The review discusses biomass and their constituents, torrefaction pre-treatment, briquetting of torrefied biomass, the parameters influencing the quality, behaviour and applications of torrefied briquettes, and way forward in the briquetting sector in the developing world.

## Introduction

Clean and sustainable fuel programmes to meet the energy needs and reduce the greenhouse gas (GHG) emissions have dominated the alternative energy and global warming space. This has driven the attention towards the non-fossil sources of fuel in developing countries. Renewable energy from solar or wind for instance, is becoming increasingly popular and could replace fossil energy resources. However, these non-fossil energy sources depend on some weather factors that are at times out of control, thereby making reliance on the alternative fossil related fuels for grid power generation inevitable (Nunes and Matias, 2020). Lignocellulosic biomass has been identified as a viable alternative bioresource capable of solving these challenges due to its abundance, renewability, clean nature, and reduction of greenhouse gases (Sher et al. 2020; Lateef and Ogunsuyi, 2021).

Despite the large prevalence of energy from bioresources in the past times, its contribution and utilization for fuel in domestic and industrial sectors remain insignificant due to its moisture variation, high volatile matter, fibrous structure, low heating value, non-uniform particle size, and low bulk and energy density (Patel et al. 2011; Hamid et al. 2016). These challenges adversely affect the handling, storage, and transportation logistics. Other key issues, such as high levels of alkali metals and halogens that could cause corrosion, fouling and slagging engines, causing startling and costly closures, have negatively impacted the use of lignocellulosic biomass for fuel (Nunes et al. 2014, 2016). Due to the contrast in the physico-chemical properties of biomass and coal, the generally utilized coal combustion chamber is non-functional for biomass fuels. This is due to the non-conformity in the design of coal conversion plant and the huge capital outlay to adapt it for biomass fuels (Nunes et al. 2014, 2016).

Meanwhile, several studies have been carried out on fuel enhancement potential of the pre-treatment of biomass and other lignocellulosic materials (Ibeto et al. 2017; Sher et al. 2020; Chaves et al. 2021). Torrefaction, which is the thermal and chemical transformation of materials at the temperature of 200 to 300 °C in an inert atmosphere, has been used extensively to transform bioresource from agricultural waste to biocoal (Basu, 2018; Mamvura and Danha, 2020; Ong et al. 2021; Yek et al. 2021). Other biomass thermochemical conversion techniques including carbonization, pyrolysis, liquefaction, gasification, and combustion are carried out under different environment and at varied temperature range (Table 1) yielding biochar, bio-oil, syngas, a combination of these or heat energy (Yek et al. 2021; Chen et al. 2021). Though the objectives of torrefaction, pyrolysis and carbonization are different, their mechanisms are virtually similar.

**Table 1:** Biomass thermochemical conversion techniques, operating temperature range, and yields

Method	Temperature range (°C)	Environment	Expected products
Torrefaction	200–300	Inert	Biochar
Liquefaction	200–600	Inert	Biochar, bio-oil, syngas
Pyrolysis	400–800	Inert	Biochar, bio-oil, syngas
Carbonization	400–1000	Inert	Biochar
Gasification	600–1300	Steam/air/oxygen	Syngas and biochar
Combustion	900–1500	Air/oxygen	Heat energy, CO_2_

Adapted from Chen et al. (2021), Yek et al. (2021)

**Fig. 1:**
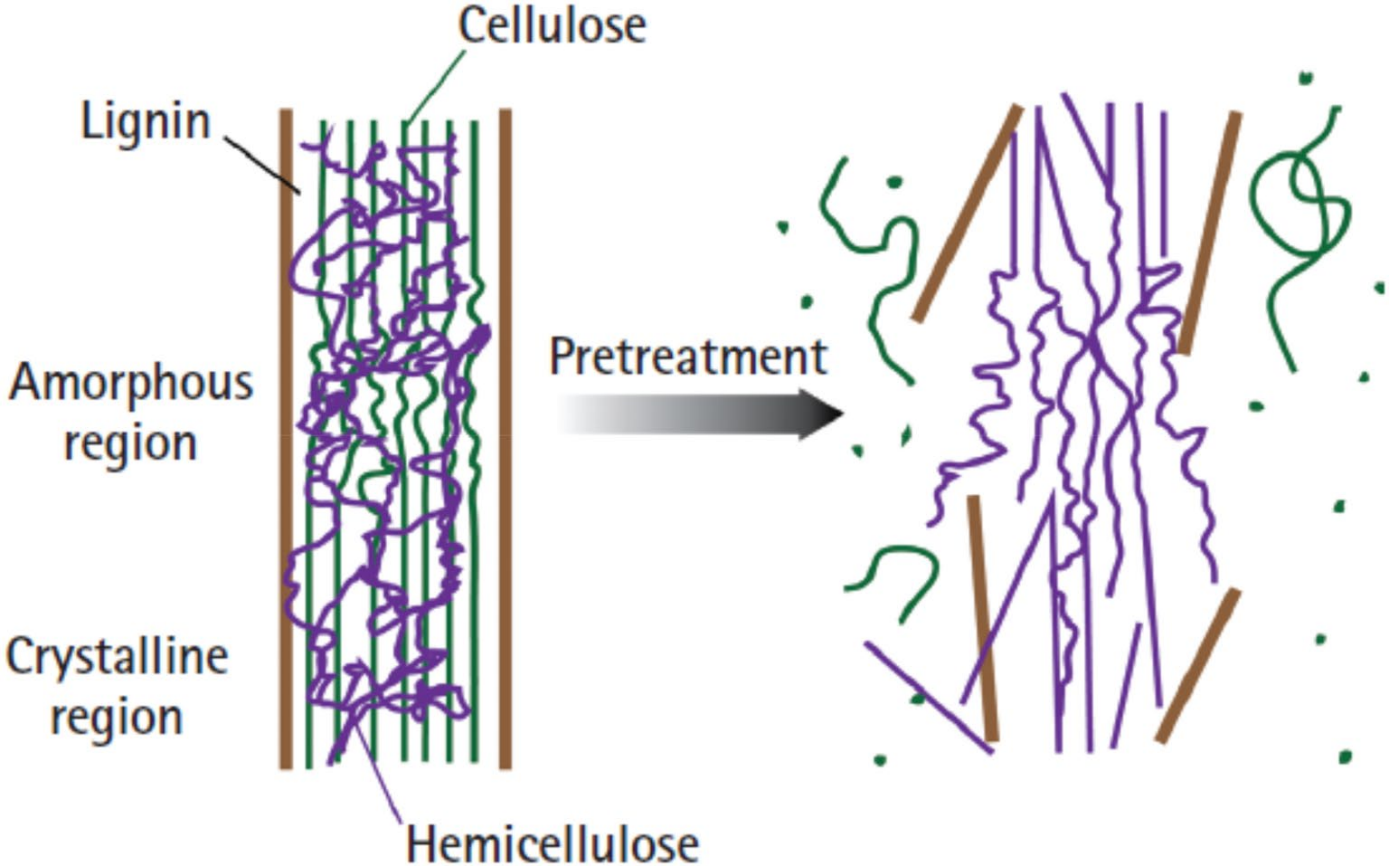
Effect of pre-treatment on the chemical structure of lignocellulosic biomass (Tumuluru et al., 2011)

Conag et al. (2017, 2018) studied the transformation of sugarcane leaves and bagasse to biocoal through torrefaction under minimized oxidative atmosphere. Akogun et al. (2022, 2022) also studied the influence of torrefaction on some tropical agricultural wastes such as cassava peel, sawdust, and cornhusk as they were transformed to useful domestic and industrial feedstock. This pre-treatment process was seen as a viable opportunity for biomass utilization that could fulfil the requirements for commercialization in line with the international standards (Faizal et al. 2018) because it enhances the heating value, fixed carbon, and carbon content of biomass. Moreover, the torrefaction process enhances the potential of raw lignocellulosic biomass by improving their grindability, hydrophobic nature and energy density (Hamid et al. 2016). These improvements are very critical to preventing moisture absorption and biological degradation during storage. Also, the reduction in the ratios of hydrogen-to-oxygen and carbon-to-oxygen in cellulose and hemicelluloses can be achieved through torrefaction (Ong et al. 2021) resulting in better quality of the lignocellulosic biomass fuel. The pre-treatment process impacts on the structure and chemical compositions (Fig. 1) of the three main components of the lignocellulosic biomass namely, cellulose (40–50%), hemicelluloses (25–35%), and lignin (15–20%). The thermal decomposition of the hemicellulose into char, non-condensable gases and volatiles occurs between 220 and 315 °C, while that of cellulose is in the range of 315–400 °C. The devolatilization of lignin takes place quite over a large temperature range of 160–900 °C and hence has better stability and yields more char than the other components during torrefaction (Shahbaz et al. 2020; Ong et al. 2021). There is a wide difference in the compositions of biomass based on the type and source which informed their different behaviours to pre-treatment (Saleh et al. 2013).

**Fig. 2:**
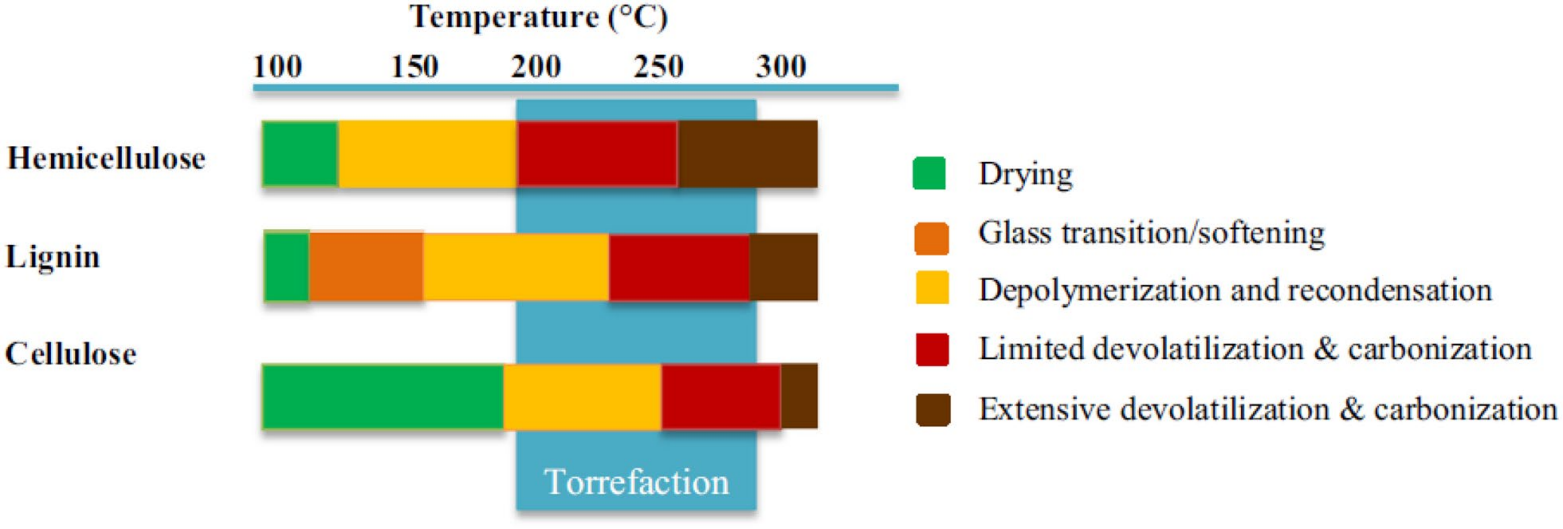
Reaction of biomass components at different torrefaction temperatures (Christoforou and Fokaides, 2016)

Olugbade and Ojo (2020) reported that depending on the applied temperature and biomass type, there are five complex steps in torrefaction pre-treatment. The pre-treatment of lignocellulosic biomass starts with non-reactive removal of moisture or drying followed by melting and softening leading to depolymerization and recondensation, followed by limited devolatilization and carbonization, while extensive devolatilization and carbonization is the last step, as shown in Fig. 2.

Handling, transportation, and storage of torrefied products in their loose form could be problematic; densification therefore becomes an avenue for better logistics and end-use applications (Gendek et al. 2018; Ibitoye et al., 2021). Briquetting transforms wastes to fuel of high value for domestic and industrial usage. Meanwhile, there is paucity of details on the update on the parameters that influence the performance of torrefied briquettes from agricultural wastes.

In the last few years, the number of review publications which focused on torrefied lignocellulosic biomass has consistently been on the increase on a yearly basis due to the growing research interest in this area of study. A number of studies have broadly reviewed different aspects of torrefaction and biomass technologies, including biomass torrefaction for solid biofuels production (Olugbade and Ojo, 2020), torrefaction technologies for the processing of biomass (Acharya et al. 2012); biomass feedstock, processing characteristics and the economics of densifying biomass (Bajwa et al. 2018). Though many of these review papers are centred on torrefied biomass, however, reviews on the state of research on the parameters that influence the quality and behaviour of torrefied briquettes from agricultural wastes is scarce in literature.

The aim of this paper is therefore to provide information on the recent development on torrefied briquettes vis-à-vis: lignocellulosic biomass and their constituents, torrefaction pre-treatment prior to its briquetting, briquetting of torrefied biomass, the parameters influencing the physico-chemical quality of torrefied briquettes, torrefaction behaviour of briquettes, applications of torrefied briquettes, challenges, and way forward in the briquetting technology sector.

## Biomass and its constituents

**Fig. 3:**
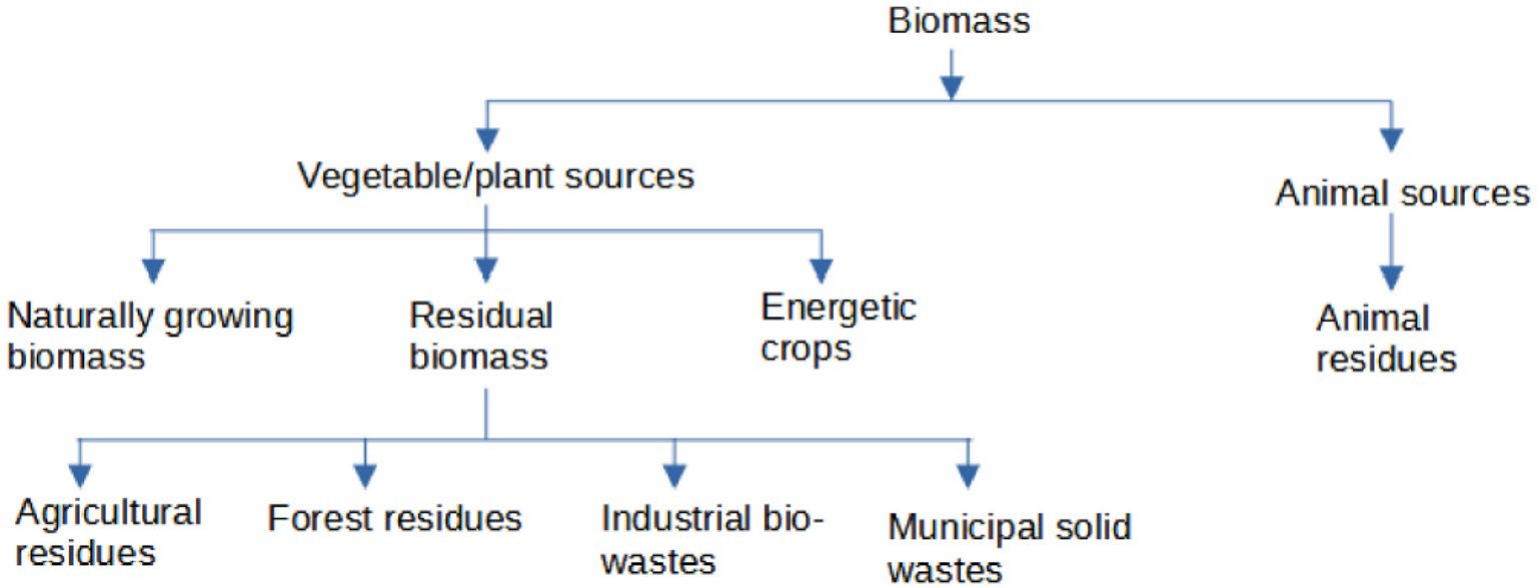
Biomass classification (Roberts et al., 2015)

Biomass is a material of biological origin which has become an important, most common, and readily available alternative renewable energy source due to its abundance, clean, bioenergy potential, low greenhouse gas generation, and low cost of production (Sher et al. 2020). Hence, it has attracted world-wide interest. Raw biomass is usually regarded as an inferior fuel when compared with modern fossil hydrocarbons such as coal (Adeleke et al. 2021). The agricultural waste biomass is classified based on its sources into plant and animal residues as shown in Fig. 3. Plant resources are the most abundant, promising, under-utilized, and cost-effective biological resources for fuel (Obi, 2015). Lignocellulosic biomass from plant resources could be converted to solid, liquid, or gaseous fuels through pyrolysis (Lee et al. 2016), torrefaction (Wallace et al. 2019; Waheed and Akogun, 2021), anaerobic digestion (Okuo et al. 2016), hydrothermal carbonization (Xu et al. 2018; Zhang et al. 2021); fermentation (Sadh et al. 2018), enzymatic hydrolysis (Chundawat et al. 2011) densification (Ibitoye et al. 2021), gasification and steam reforming (Ajala et al. 2021), and transesterification reactions (Alamu et al. 2008).

The major worldwide-known compositions of lignocellulosic biomass are hemicellulose (20–40 wt %), cellulose (40–60 wt %) and lignin (10–25 wt %) (Chen et al. 2015; Bajwa et al. 2018). Cellulose is an insoluble substance which is the main constituent of a plant cell wall and of vegetable fibres. It is a beta 1,4 glucose polymer. Hemicellulose, the most reactive at the torrefaction temperature below 250 °C, has complex carbohydrate polymer with low molecular weight. It consists of D-xylose, D-galacturonic, D-galactose, D-glucose, L-arabinose, 4-O-methylglucuronic, D-mannose and D-glucuronic acids (Acharya et al. 2012). The high content of lignin in biomass has a binding impact on the high-quality briquette production (Akande and Olorunnisola, 2018). Moreover, it has dehydration challenge and difficulty in char conversion than hemicellulose or cellulose.

The lignocellulosic biomass as an environmental resource has several challenges that limits its utilization and applicability for fuel and chemical production. The limitations include its high volatile matter, low bulk and energy density, high alkali and moisture contents, hygroscopic and poor combustion behaviour, its fibrous nature, and excess air pollution leading to low combustion efficiency (Patel et al. 2011; Martin et al. 2015).

These limitations severely impart the combustion performances due to poor logistics including handling, transportation, and storage of biomass (Conag et al. 2018). However, biomass efficiency and utilization can be enhanced through drying, size reduction, torrefaction, and briquetting (Kopczynski et al. 2017). The recent developments in biomass studies show that torrefaction pre-treatment offers promising results for the conversion of biomass into bioenergy on largescale for sustainable domestic and industrial energy applications.

## Torrefaction pre-treatment of biomass for briquette production

Torrefaction is the process of converting lignocellulosic biomass into carbon-rich substance through heating (Mukhtar et al. 2019). It is the thermal and chemical process by which biomass is treated at atmospheric pressure in an inert atmosphere or at minimum amount of oxygen to avoid spontaneous combustion (Conag et al. 2017, 2018). Literature shows that the temperature range used for torrefaction process differ widely between 150–350 °C due to the differences in biomass feedstocks (Zhang et al. 2018a, b; Chaves et al. 2021). The energy requirement for torrefaction is lower than that of pyrolysis due to the lower temperature requirement in the former (200–300 °C) as against a high temperature range (400–900 °C) in the latter (Azam et al., 2015, Saeed et al., 2015). Torrefaction can be carried out in the absence of oxygen (Garba et al., 2017) or at minimized oxidative environment (Conag et al. 2017, 2018). Furthermore, torrefaction is also a pre-treatment process carried out at less than 50 °C/min and retention time of 10 to 120 min (Balogun et al. 2014; Ibeto et al. 2016). Agricultural waste behaves differently to thermal treatment because of their origin, types, and properties (Akogun and Waheed, 2022). The three basic observed phases in the process of torrefaction of raw biomass are drying (moisture removal), removal of volatiles and tars, and production of solid biomass at high torrefaction temperature.

There are two types of torrefactions: dry and wet torrefaction. Dry torrefaction involves the thermal decomposition of dried biomass (i.e. < 15% moisture) mainly into hemicellulose due to limited supply of oxygen at a temperature range 200–350 °C (Nhuchhen et al. 2014). Wet torrefaction on the other hand is a sustainable subcritical water pre-treatment technology to enhance moist biomass into solid fuel (Zhang et al. 2021). Generally, torrefaction process results in improved qualities of biomass characterized by low moisture levels, high energy and density, and better hydrophobic behaviour (Hamid et al. 2016). During torrefaction, the appearance and handling properties of the raw biomass are changed while the resultant darker fuel has higher heating value, better energy density, altered the fibrous behaviour, improved hydrophobicity, and ease of grinding (Garba et al. 2017).

Moreover, there is an alteration in the carbon (C), hydrogen (H) and oxygen (O) constituents of biomass. The O/C and H/C ratios reduce due to the reduction in oxygen and hydrogen contents relative to the carbon content (Akogun and Waheed, 2019; Nunes et al. 2021) which is an indication of the maximized mass yield and energy of biomass (Chen et al. 2015). Moreover, torrefaction also results in reducing volatiles and moisture content through heating (Portilho et al. 2020; Chaves et al, 2021). It helps to ameliorate the limitations of raw feedstocks for briquette production and upscale their qualities (Nhuchhen et al. 2014). During torrefaction process, torrefied solids (chars) containing high carbon content are produced through biomass decomposition. The weight loss kinetics of torrefied biomass by increase in temperature was explored in literature (Garba et al. 2017). The kinetics of torrefaction occurs first and faster at the decomposition of hemicellulose, followed by the cellulose which occurs slowly, and this significantly contributes towards the overall mass yield of torrefied fuel (Dhaundiyal and Hanon, 2018). Since torrefied fuel is produced in a loose form, a viable technique of improving their handling and logistic issues is through briquetting.

## Briquetting of torrefied biomass

**Fig. 4:**
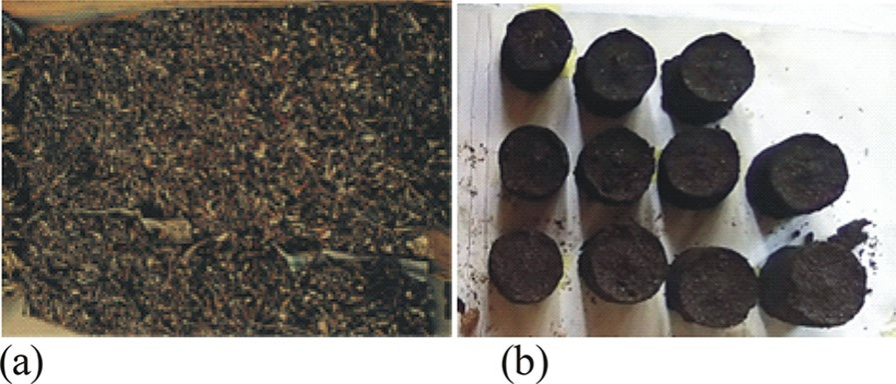
**a** Dried cassava peel (Akogun et al., 2020); **b** torrefied briquette sample (Waheed and Akogun, 2021)

A huge quantity of agricultural wastes is generated globally and their attractiveness as a bioresource is increasing (Langsdorf et al. 2021). Unfortunately, the direct burning of these residues is inefficient, as it poses environmental pollution and hazards (Oladeji, 2012) and contributes to global warming. Other challenges of biomass utilization for fuel include the handling, transportation, and storage difficulties (Gendek et al. 2018; Langsdorf et al. 2021). Powdered torrefied product could lead to air pollution and pose respiratory challenge when inhaled. A sure way to ameliorating some of these stated limitations is through densification to a product of higher density and quality (Gendek et al. 2018). A study by Waheed and Akogun (2020) revealed that biomass thermally treated before compaction, can produce physically strong fuels with enhanced thermal characteristics (Fig. 4). This is attributed to the release of lignin component present in biomass when subjected to heat. The appropriate moisture preconditioning of lignin enhances particles bonding and produces physically strong fuels (Tumuluru et al. 2011; Han et al. 2013; Jiang et al. 2014).

Briquetting is an efficient alternative of biomass conversion process through which agricultural wastes are compacted into highly dense and durable fuel. Briquettes may be produced with or without binders into different shapes or forms such as square and cylindrical by compression into usable feedstock for many applications (Fuad et al. 2020).

Meanwhile, briquettes produced from the densification of torrefied products have a low moisture content and volatile emission, less biological activity, high fixed carbon content and heating value (Keeratiisariyakul et al. 2019) thus enabling an increase in the heating value up to 20% (Akogun et al. 2022). The torrefied briquettes retain some portion of the original mechanical characteristics used in several combustion applications (Oladeji, 2012). The appearance of the torrefied briquettes depends on the type of feedstock, applied pressure, retention time, level of compactness, particle size and the shape of mould used. Generally, there is improvement in the physico-mechanical properties of torrefied fuel compared to those of raw biomass in loose form (Waheed and Akogun, 2020). Table 2 compares the properties of raw biomass, torrefied briquettes, and coal. Combined torrefaction and briquetting is still being developed as a pre-treatment technology for biomass at research stage. The readiness level will rely on several factors, such as market price, technical possibilities, government intervention, and the prevailing trends of bioenergy development but the prospect is on the high side putting these factors into consideration. Meanwhile, to the best of the authors’ knowledge, there is no known major commercial/industrial scale production of the torrefied briquettes in practice but there are experimental scale plants for demonstration purpose (Ong et al. 2021). Mostly, torrefied briquettes are used only for testing purpose. The biggest known demonstration torrefaction plant with a capacity of 0.8 million tons/year is situated in Mississippi (USA) (Proskurina et al. 2017).

**Table 2:** Properties of raw biomass, torrefied briquettes, and coal

Properties	Raw biomass	Torrefied briquettes	Coal
Moisture content (MC)	High (> 15 wt%)	Low (< 5%)	Low (< 5%)
Volatile matter (VM)	High (65–88 wt%)	Low (35–45%)	Low (0.9–50%)
Fixed carbon (FC)	Low (0.5–20 wt%)	Moderate (20–45%)	High (46–92%)
Carbon	39–50 wt%	45–68 wt%	64–92 wt%
Oxygen	37–50 wt%	11–45 wt%	1–25 wt%
Atomic O/C ratio	0.4–0.8	0.1–0.7	–
Atomic H/C ratio	1.2–2.0	0.7–1.6	–
Volumetric energy density (GJ/m^3^)	2.0–3.0	15.0–18.7	18.4–23.8
Handling and logistics	Low bulk density	High bulk density	Low handling requirements
CO_2_ emission	Carbon-neutral	Carbon-neutral	Fossil Fuel
Co-Firing Ratio with Coal	Low co-firing ratios due to low bulk density	Up to 50% or more	N/A
Heating value	9–12 MJ/kg	15–24 MJ/kg	23–35 MJ/kg
Dust explosibility	Average	Limited	Limited
Hydroscopic properties	Hydrophilic	Hydrophobic	Hydrophobic
Biological degradation	Yes	No	No
Transport cost	High	Low	Low

Nunes and Matias (2020), Chen et al. (2021)

## Physico-mechanical and torrefaction parameters influencing the behaviour and quality of torrefied briquettes

**Fig. 5:**
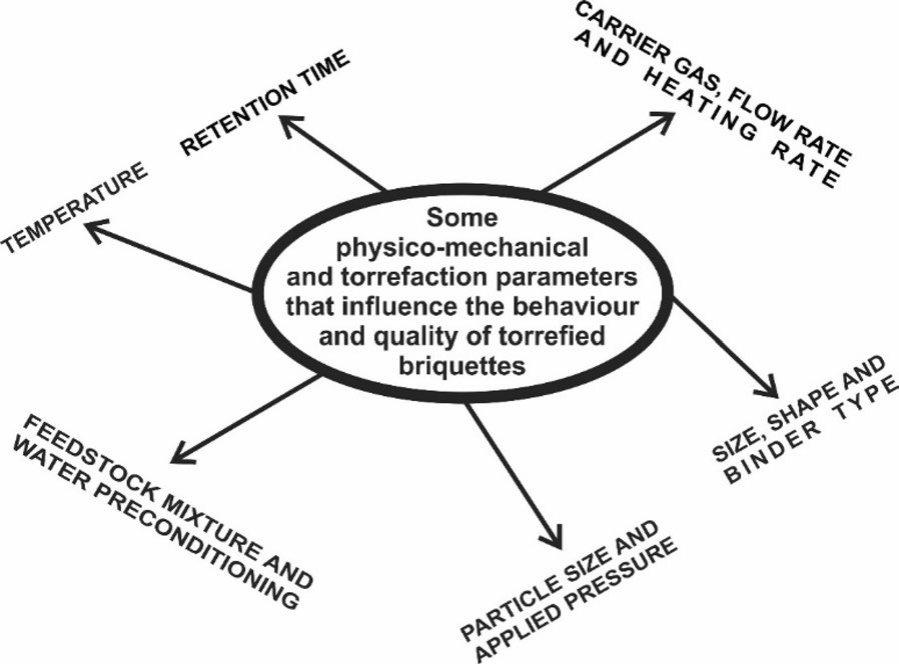
Variables that influence the properties and quality of torrefied briquettes

Some physico-mechanical and torrefaction parameters (Fig. 5) that influence the behaviour and quality of torrefied briquettes are discussed in what follows.

### Temperature

Heating to high temperature significantly influences the decomposition of biomass and their performance (Martin et al. 2015). There is the decomposition of polymer during the heating of biomass materials due to the increase in temperature (Martin et al. 2015). This is one of the several reasons why researchers have shown interest in the study of temperature effect on fuel performance (Nyakuma et al. 2015; Faizal et al. 2016). Also, the degradation of cellulose starts from 240 to 350 °C due to high resistance of its crystalline arrangement to thermal breakdown into monomers because of its strength (Tumuluru et al. 2010). Akogun et al. (2020) reported that the volatiles and moisture contents present in sawdust and cassava peel briquettes reduce with temperature increase. On the other hand, the increase in temperature produces high fixed carbon content, which results in carbonization (Portilho et al. 2020; Chaves et al., 2021). Torrefaction causes the biomass to lose low molecular weight volatiles caused by decarboxylation and moisture loss of the long polysaccharide chains (Garba et al. 2017). Thereafter, about 70% of the initial weight of biomass and about 90% of the original energy recovered is obtained as solid torrefied fuel (Prins et al. 2006). Thus, using the appropriate torrefaction temperature helps raw biomass to overcome the limitations of high-water absorption, low heating and energy–density, and high moisture content. In contrast, the combustion of raw lignocellulosic biomass produces high volatiles including tars and oil which is problematic (Mei et al. 2015). In grindability studies, the particle size distribution is improved to a great extent if the raw biomass is pre-treated through torrefaction. That is, particle size decreases significantly with the rise in torrefying temperature.

### Retention time

Retention time is the time from one section of biomass decomposition to another and depends on the type of biomass. The time from depolymerization to devolatilization is shorter for hemicellulose than for lignin and cellulose due to the higher reactivity and small temperature range of the former than those of the latter (Acharya et al. 2012). Thus, an increase in the retention time during torrefaction results in the reduction of the biomass yield and the release of more volatiles (Balogun et al. 2014). The parameters usually used during biomass torrefaction studies are temperature and retention time; but retention time has less effect compared to temperature (Martin et al. 2015). Meanwhile, during operation, these two parameters have impact on each other. For instance, Grigiante and Antolini (2014) stated that an increase in temperature from 280 °C to 310 °C led to a retention time reduction of 59 min to obtain similar mass and energy values. Thus, the temperature increment of 30 °C saved about 1 h of time. The composition of fixed carbon increased with the retention time as noted by Wang et al. (2018). Olugbade and Ojo (2020) stated that the increase in the retention time leads to an increase in the energy density and grindability. This is due to the increased disintegration of the fibre structure of biomass with retention time, even though at a minimal rate in comparison with the increase in temperature. The process of torrefaction can be done for few minutes to about 2 h. Waheed and Akogun (2020), however, stated that torrefaction retention time should be maintained within one hour. Table 3 summarizes the effect of torrefaction temperature and retention time on the results obtained for some selected lignocellulosic biomass.

**Table 3:** Summary of the torrefaction temperature, retention time and results obtained on some selected lignocellulosic biomass

Biomass	Torrefaction temperature (°C)	Retention time (mins)	Basic results	Refs.
Sugarcane bagasse	230–290	180	At 290 °C, the weight loss was more than twice faster than at 230 °C	Granados et al. (2017)
Rubber wood	150–300	120	At 150 °C, no thermal decomposition occurred however, at 250 °C, the weight loss increased when potassium concentration increased. At 300 °C, mass yield decreased when potassium concentration increased	Safar et al. (2019)
Woody biomass	225- 300	20, 30, 40	The rate of by which volatiles was given off was 4.16%/min for light, 1.80%/min (mild) and 0.70%/min (severe). Weight loss during torrefaction was described as 3–6% for light; 9–14% (mild); and 11–16% (severe)	Moya et al. (2018)
Rice husk	210–270	60	Decrease in the mass loss; decrease in differential thermogravimetric max value	Zhang et al. (2018a, b)
Rice straw	250	15	Gas-pressurized torrefaction promoted the cellulose decomposition	Tong et al. (2018)
Pine straw	250	15	Torrefaction carried out by pressurized gas gave increased char yield than atmosphere induced process	Tong et al. (2018)
Washed rice husk	250–280	20	The initial temperature for decomposition was increased. Increase in the gradual breakdown of hemicellulose and lignin	Zhang et al. (2016)
Camellia shell	260	30	At low temperature, there is decomposition of the structure and part of organic compounds; the maximum mass loss shifted from 286–346 °C in dry torrefaction while there is the decomposition of hemicellulose in wet torrefaction	Xu et al. (2018)
Bamboo	230–250	60	O_2_ gas was more effective to remove hemicellulose than CO_2_	Su et al. (2018)
Cornhusk, cassava peel and sawdust	200–300	60	Mass loss increased with increase in torrefaction temperature at 300 °C than at 200 °C	Akogun and Waheed (2022)

### Carrier gas, flow rate and heating rate

In recent times, some researchers have studied the influence of various carrier gases, such as nitrogen gas, flue gas, CO_2_, and air, on the torrefied products (Mei et al. 2015). In the study of the effect of torrefaction on palm kernel shell (PKS) briquettes by Fuad et al. (2020), it was established that the increase in the flow rate of nitrogen gas from 1000 to 2000 mL/min led to the increase in density yield from 73 to 79%, and a slight decrease to 75% at the flow rate of 3000 mL/min. In the same vein, the increase in nitrogen flow rate produced an increase in the mass yield from 60 to 64% while the energy yield was found to be within the range of 73 to 75%. On the contrary, Nyakuma et al. (2019) claimed that the increase in nitrogen flow rate during torrefaction gave an insignificant change of yields and a more unreactive atmosphere. Hernández et al. (2017) torrefied sewage sludge under CO_2_ and N_2_ atmosphere in a thermogravimetric-Fourier transform infrared spectroscopy experiment. Their results show that the use of CO_2_ during torrefaction resulted in an increased decomposition rate while the peak of gradual breakdown was shifted by less than 7 °C.

Rousset et al. (2012) studied the effect of different O_2_ concentrations on varying torrefaction temperature using *Eucalyptus grandis* as feedstock. It was revealed that at high temperature of 280 °C, the effects of the O_2_ on the solid yield and char characteristics were more evident. Moreover, Batidzirai et al. (2013) reported that during torrefaction, slow heating rate (< 50 °C/min) promotes maximum solid yield. The overall agreement from literature is that the carrier gas, gas flow rate and heating rate have effects on the density yield, mass yield, energy yield and rate of decomposition of biomass depending on the biomass type, type of carrier gas used and the flow rate.

### Feedstock mixing and water preconditioning

The characteristics of mixed raw agricultural wastes was studied by some authors such as Mitchual et al. (2013) and Kpalo et al. (2021) but the qualities of the produced briquettes were not suitable for heating applications in engines designed for coal combustion. This informed the need to carry out studies on blending raw agricultural wastes with coal (Ohm et al. 2015; Adeleke et al. 2021). Unfortunately, the aftermath of coal usage as feedstock is not environmentally acceptable nor friendly. It is therefore needful to carry out more studies on the thermochemical conversion technique called torrefaction pre-treatment or mild pyrolysis on agricultural wastes, whose properties are close to coal (Garba et al. 2017) to overcome the challenges of blending raw biomass feedstock and coal. The blending of two torrefied agricultural wastes with another was recommended for application (Akogun et al. 2022). For the appropriate deployment of torrefied products for usage therefore, there is a need to enhance their handling and density via briquette production (Li et al. 2012). However, torrefied waste samples are usually difficult to densify in comparison with raw ones under the same briquetting conditions (Faizal et al. 2018) when not pre-treated with deionized water (Bai et al. 2017).

A study by Akogun et al. (2022) revealed that the addition of cornhusk to sawdust both in the raw and torrefied form caused the density of the briquette produced to increase between 12–19% depending on the mixture level, compared with the briquettes composed of 100% of the torrefied sawdust. This increase was attributed to the sprinkling of distilled water on the biomass samples before densification as observed in the work of Bazargan et al. (2014). The enhanced binding ability, compactness, and increased density value of the torrefied fuel in comparison with the raw ones were caused by the increase in the lignin value through the pre-heating of the feedstock and water preconditioning of the torrefied products before they were compressed (Bai et al. 2017). The plasticity of the biochar particles decreased with torrefaction leading to higher energy consumption during pelletization which could be reduced by water preconditioning (Han et al. 2013). The hydrogen bonding capability provided by the deionized water preconditioning therefore enhances the plasticity of the torrefied products and decreases the energy consumption. The sprinkling of the deionized water on torrefied samples prior to densification was said to also improve the shatter index (Tumuluru et al. 2011).

### Particle size and applied pressure

The particle size distribution is the number of particles according to size retained and sorted. That is, it describes the size of particles of feedstock as determined. Chipping reduces the particle size of biomass to 10–30 mm while grinding and milling reduces it up to 0.2 mm (Kumar and Sharma, 2017). The strength properties of briquettes such as density and durability are inversely proportional to particle size since smaller particles have greater contact surface area during densification. The higher heating value of the briquettes was found to increase with the reduction in the particle size. Oladeji and Enweremadu (2012) examined the effects of varying particle sizes of 4.70, 2.40 and 0.60 mm on some characteristics of corncob briquettes. The study revealed that corncob with 0.60 mm particle size gave the best positive attributes than the other two-particle sizes. The study also revealed that the density and durability of a fuel is enhanced with the reduction of the particle size of the feedstock. On the other hand, the applied pressure also affects the physico-mechanical properties of briquettes. The increase in the applied pressure and decrease in particle sizes of a feedstock yields briquettes with improved physical and mechanical properties as can be seen in Table 4.

**Table 4:** Effect of particle size and applied pressure on some physical and mechanical properties of briquettes produced from selected agricultural and forestry wastes

Feedstock	Particle size	Applied pressure (MPa)	Relaxed density (kg/m^3^)	Compressed density (kg/m^3^)	Durability index (%)	Compressive strength (MPa)
^a^Corn cob	˂4.00 mm	200	1018.40	–	99.80	40.40
^a^Corn cob	˂4.00 mm	150	949.30	–	63.20	30.50
^b^Groundnut shell	600.00–850.00 μm	–	151.00	411.01	93.52	–
^c^Coal-rice husk	–	20–45	1055.4–1663.50	–	61.87–138.50 cm	0.116–0.766
^a^Bean straw	˂4.00 mm	200	1153.20	–	99.80	99.60
^a^Bean straw	˂4.00 mm	150	1063.00	–	99.80	92.60
^d^Pine	6.00 mm	21	938.54	–	93.91	2.81
^d^Spruce	6.00 mm	21	1078.39	–	97.87	9.69
^d^Larch	6.00 mm	21	944.38	–	87.58	4.16
^e^*C. pentandra*	< 1.00 mm)	50	716.00	–	–	44.58
^e^*T. scleroxylon*	< 1.00 mm)	50	632.00	–	–	30.56
^e^*A. robusta*	< 1.00 mm)	50	695.00	–	–	26.63
^e^*T. superba*	< 1.00 mm)	50	727.00	–	–	18.92
^e^*P. Africana*	< 1.00 mm)	50	741.00	–	–	21.14
^e^*C. mildbreadii*	< 1.00 mm)	50	706.00	–	–	12.45

^a^Okot et al. (2019)

^b^Oyelaran et al. (2014)

^c^Markson et al. (2013)

^d^Gendeka et al. (2018)

^e^Mitchual et al. (2013)

### Size, shape, and binder

The shape and dimension of a briquette have an influence on its density, handling characteristics and speed of combustion (Tabares et al. 2000). For instance, the work of Akogun and Waheed (2022) shows that the cylindrical shaped briquettes have enhanced density, compressive strength, and shatter index than the square-shaped ones. However, the square-shaped briquettes would occupy less void space than the cylindrical ones during storage. Moreover, Tabares et al. (2000) said that square-shaped briquettes have greater mass loss than cylindrical ones during combustion because their burning usually start at the edges. Meanwhile, briquettes with large diameter tend to burn slowly. It was then concluded that the briquette behaviour could be predicted and modified by varying the diameter and the feedstock type (Tabares et al. 2000). The dimension of briquette influences the type and size of stove that can be used for the briquettes to fit in well inside the combustion chamber of the stove. However, the strength, durability and water absorption capacity of densified biomass are very important indices for handling, and they can be improved through the addition of binding materials (Fuad et al. 2020). Binders are classified into three; organic, inorganic, and compound, based on their composition (Kivumbi et al. 2021).

Organic binders have high combustion efficiency for heating applications than inorganic binders which though have strong bonding strength do not have good commercial applications because of their good hydrophilicity (Shu et al. 2012). Compound binders comprising the combination of organic and inorganic binders are better considered to take advantage of the limitations of organic and inorganic binders, thus yielding briquettes with high mechanical strength and thermal stability (Zhang et al. 2014). Examples of organic based binders are water hyacinth, gum Arabic, cassava starch, molasses, lignin, etc. Inorganic binders include cement, clay, limestone, calcium oxide, bentonite, iron oxide, etc., while the compound-based binders are resin and starch, bentonite and starch, pitch, and molasses, etc. The selection of binders depends on the environmental friendliness, availability, low emissions, low cost, desired bonding strength, sustainability, and economic availability (Zhang et al. 2018a, b).

## Behaviour of torrefied briquette in relation to raw ones

### Combustion indices

The combustion indices are normally used to present a clearer understanding in assessing fuel from pre-treated raw feedstock to ensure that the performance of the coal-powered generation system is not jeopardized (Ohm et al. 2015). The use of the pre-treated biomass as fuel would be best applicable if its characteristics is like that of coal. The changes in the combustion indices including fuel ratio (*FR*), volatile ignitability (*VI*) and combustibility index (*CI*) defined in Eqns. (1–3) are usually determined to assess the fuel quality and performance of the torrefied briquettes produced from agricultural wastes:

1 $$Fuel \,ratio, FR= \frac{{FC}_{db}}{{VM}_{db}}$$

2 $$Combustibility index, CI \left(\frac{MJ}{kg}\right)= \frac{{HV}_{db}}{105FR}\times \left(115-{Ash}_{db}\right)$$

3 $$Volatile ignitability, VI \left(\frac{MJ}{kg}\right)=\left[\frac{{HV}_{db}-0.338{FC}_{db}}{{VM}_{db}+ {MC}_{db}}\right]\times 100,$$

where the subscript db denotes dry basis, *FC* fixed carbon, *VM* volatile matter, *HV* heating value, and *MC* moisture content.

Waheed and Akogun (2020) studied the combustion indices of raw cornhusk and cassava peel briquette blends, and they reported that the fuel ratio of the raw briquette blend was less than that of coal; hence, it cannot be used as fuel in coal engines. Fortunately, the briquettes produced with the blend of the biomass feedstocks torrefied at 300 °C yielded higher values of the fuel ratio and volatile ignitability, and lower value of combustibility index over those for the raw ones. Similar trends on the effects of torrefaction on these indices were also reported by Ohm et al. (2015). Meanwhile, fuels with combustibility index value of 12.56–23.03 MJ/kg are applicable for combustion in coal thermal engines (Conag et al. 2017; 2018). Table 5 presents the combustibility index of some feedstocks as influenced by torrefaction. The fuel ratio, volatile ignitability and combustibility index of briquette torrefied at 300 °C for at least 1 h meets the standard values of 0.5 ≤ *FR* ≤ 2.0, *VI* ≥ 14.5 MJ/kg and 12 ≤ *CI* ≤ 23 MJ/kg and the fuels are applicable for joint combustion with coal in coal engines (Akogun and Waheed, 2022).

**Table 5:** Effect of torrefaction on the combustion indices of briquettes

Feedstock	Torrefaction temperature, retention time and device	Raw Feedstock			Effect of torrefaction on the combustion indices of some raw feedstock		
		FR	VI (MJ/kg)	CI (MJ/kg)	FR	VI (MJ/kg)	CI (MJ/kg)
^a^Cornhusk	300 °C/60 min, electric furnace	0.26	15.72	54.15	0.73	27.58	25.53
^a^Sawdust	300 °C/60 min, electric furnace	0.25	15.77	60.47	0.82	28.80	24.97
^a^Cassava peel	300 °C/60 min, electric furnace	0.28	16.02	46.47	0.86	30.17	17.68
^b^Palm kernel shell	350 °C/30 min, electric furnace	0.36	–	14.00	0.40		13.50
^b^Bagasse	350 °C/30 min, electric furnace	0.21	–	22.00	0.40		13.00
^b^Waste wood	350 °C/30 min, electric furnace	0.20	–	27.00	0.49		11.00
^c^Sugarcane bagasse	350 °C/75 min, furnace	0.22	12.55 ± 1.51	82.51 ± 7.80	1.68 ± 0.01	16.27 ± 0.07	16.04 ± 0.09

*FR* fuel ratio, *CI* combustibility index, *VI* volatile ignitability

^a^Akogun and Waheed (2022)

^b^Ohm et al. (2015)

^c^Conag et al. (2017)

### Compositional constituents

The compositional constituents of a lignocellulosic biomass fuel consist of hemicellulose, cellulose, lignin, and some extractives (Kumar and Sharma, 2017). The amount of hemicellulose and extractives in biomass samples decreased when torrefied, rendering them susceptible to disintegration and decomposition due to the presence of heteroatoms and loose structures in these components (Zhang et al. 2016; Su et al. 2018). The cellulose contents of the biomass samples are also reduced, leading to biomass decomposition through torrefaction effect (Tong et al. 2018). The content of lignin in biomass are however increased due to torrefaction (Sumaira et al. 2019). The possible reason for this higher value in lignin after torrefaction could be due to the decreased contents of hemicellulose, cellulose, and extractives in the torrefied samples. Severe torrefaction of biomass usually takes place at temperature above 270 °C and is characterized by a noticeable effect on the lignin and cellulose and whereas light torrefaction is characterized by a marked decomposition of hemicellulose in which lignin and cellulose are slightly affected and usually occurs below 240 °C. Hemicellulose and cellulose produce mainly volatiles while lignin produces mainly char (Zhao et al. 2017) and improves densification properties due to its thermoplastic behaviour. Extractives act as lubricants during compression. It also prevents strong bond formation by creating a layer between particles (Huang et al. 2017). Moreover, the hydroxyl group in hemicellulose and lignin helps in particle bonding through formation of hydrogen bonds (Huang et al. 2017).

At high temperature, moisture evaporates and hydrolyses cellulose, hemicellulose, and lignin partially to lower molecular mass products which act as binders and improve the mechanical strength of briquettes (Li et al. 2012). Temperature minimizes relaxation and improves the degree of densification, facilitating the release of lignin, cellulose and hemicellulose which are natural binders that form solid bridges upon cooling thereby increasing the mechanical strength and density (Lee et al. 2013). Hemicellulose and cellulose partially or fully decompose at the tested range of 200–300 °C and produce H_2_O, CO_2_, CO, and some organic compounds such as acetic acid, phenol, and furfural (Poudel et al. 2015). The intensity of O–H decreased with increasing temperature due to decomposition of lignocellulosic components especially hemicellulose (Chen et al. 2015; Mei et al., 2016). Torrefaction weakens the fibre structure in biomass as hemicellulose, cellulose and lignin have different thermal stabilities which makes biomass materials more brittle and fragile (Chen et al. 2015; Zheng et al. 2017). The partial decomposition of biomass results in the changes in its properties and this enhances its briquetting potential and fuel capacity.

### Heating value

The measurement of the value of heat or energy released by a fuel during complete combustion is obtained using a bomb calorimeter. The increase in the torrefaction temperature leads to the enhanced heating value of torrefied fuel due to the increase in carbon and fixed carbon (Lateef and Ogunsuyi, 2021). For instance, the heating value of palm kernel shell briquettes increased from 17.88 to 22.16 MJ/kg due to torrefaction (Fuad et al. 2020). Garba et al. (2017) reported that the heating values of corn cob, rice husk and groundnut fuel were enhanced by factors of 1.26, 1.32, and 1.18, respectively, at the temperature of 300 °C. Conag et al. (2017, 2018) also reported an increased trend in the heating value when sugarcane leaves and bagasse were torrefied. Torrefaction is therefore seen as a means of eliminating some challenges attributed to raw biomass as it significantly upgrades the fixed carbon and heating value and reduces the volatile matter.

### Proximate properties

The proximate properties of a fuel are determined in terms of the volatile matter (VM), moisture content (MC), fixed carbon (FC), and ash content (AC). Some other elements such as magnesium, potassium and chlorine are also found in the lignocellulosic biomass (Tripathi et al. 2016). The value of the fixed carbon content is found to increase while that of the volatile matter reduces when the torrefaction temperature and retention time are increased (Conag et al. 2018; Mukhtar et al. 2019). Moreover, with the increase in the torrefaction temperature, the carbon content increases, and the oxygen content decreases which culminate in the increase in the heating value of briquettes (Conag et al. 2017, 2018) as can be seen in Tables 6 and 7. Meanwhile, the ash content (AC) increases with the increase in the torrefaction temperature (Patel et al. 2011; Conag et al. 2018). For instance, the ash content increased slightly from 3.75 to 4.5% when raw cornhusk briquettes were torrefied at 300 °C. Moreover, the ash content for raw sawdust briquettes increased from 1.65 to 1.75% when torrefied at 300 °C and that for the cassava peel briquettes from 9.85 to 14.95% (Akogun et al. 2022). Meanwhile, briquettes with the ash content less than 20% are suitable for domestic and industrial applications including co-firing with coal (Patel et al. 2011; Conag et al. 2018). The need for the discharge of more ash from the combustion chamber increases with the ash content.

**Table 6:** Effect of torrefaction on the proximate properties of agricultural feedstocks

Feedstock	Torrefaction temperature, retention time and device used	Proximate properties				Effect of torrefaction on the proximate properties of the feedstock			
		VM%	MC%	FC%	AC%	VM%	MC%	FC%	AC%
^a^Sugarcane bagasse	300 °C/90 min, furnace	82.25	3.31	16.38	1.36	27.10	3.79	68.52	4.39
^b^*Coffee arabica*	300 °C/180 min, electric oven	78.07	–	19.37	2.56	65.53	–	31.12	3.35
^b^*Eucalyptus* spp^.^	300 °C/180 min, electric oven	86.75	–	12.93	0.32	76.07	–	23.45	0.48
^c^Cassava peel	260 °C/20 min, programmable muffle furnace	67.30	9.00	12.00	3.50	60.80	1.00	29.90	3.90
^c^Coconut shell	260 °C/20 min, programmable muffle furnace	71.80	11.50	20.50	0.70	71.80	1.50	34.90	1.40
^*d*^*Phyllostachys aurea*	220 °C/60 min, Macro Thermogravimetric reactor	83.70	10.99	15.28	1.01	81.64	2.73	17.35	1.02
^e^Cornhusk	300/60 min, furnace	70.50	8.50	18.25	3.75	50.50	8.30	36.70	4.50
^e^Sawdust	250/60 min, furnace	72.60	8.90	17.85	1.65	61.00	8.80	28.70	2.50
^f^Logging residue	350/30 min, electric furnace	69.39	10.12	19.74	0.75	63.62	2.73	27.20	6.45

*VM* volatile matter, *MC* moisture content, *FC* fixed carbon, *AC* ash content

^a^Nunes et al. (2021)

^b^Portilho et al. (2020)

^c^Ibeto et al., (2017)

^d^Chaves et al. (2021)

^e^Akogun and Waheed (2022)

^f^Ohm et al. (2015)

**Table 7:** Effect of torrefaction on the elemental constituents and the heating values of some agricultural wastes, and blends

Feedstock	Torrefaction temperature, retention time and device used	Elemental composition and heating value						Effect of torrefaction on the elemental composition and heating value					
		C%	H%	O%	S%	N%	HV (MJ/kg)	C%	H%	O%	S%	N%	HV (MJ/kg)
^a^Groundnut shell	300 °C/60 min, glass tube pyrolyzer	45.32	6.03	43.54		0.51	18.42	55.00	5.29	37.7	–	0.51	22.66
^a^Rice husk	300 °C/60 min, glass tube pyrolyzer	43.83	6.76	46.07	–	0.93	15.81	53.56	5.81	39.45	–	0.77	20.87
^b^Coconut shell	300 °C/20 min, furnace	46.60	7.10	41.80	–	0.32	14.10	68.60	1.89	12.80	–	0.65	26.00
^c^Sugarcane bagasse	300 °C/90 min, furnace	47.30	6.56	45.54	–	0.60	19.45	78.60	6.46	14.56	–	0.38	33.45
^c^Cashew nutshells	300 °C/90 min, furnace	53.50	6.39	39.68	–	0.44	22.05	69.50	5.19	24.35	–	0.96	27.69
^d^Soybean straw	350 °C, 45 min, tube furnace	44.30	5.80	45.20	0.10	0.70	16.70	58.70	4.10	26.50	0.10	1.10	21.10
^e^Logging residue	300 °C, 30 min, electric furnace	51.59	6.52	40.96	0.11	0.83	21.19	54.48	5.32	39.01	0.04	1.15	22.03

*C* carbon content, *H* hydrogen content, *O* oxygen content, *S* sulphur content, *N* nitrogen content, *HV* heating value

^a^Garba et al. (2017)

^b^Ibeto et al. (2016)

^c^Nunes et al. (2021)

^d^Zhang et al. (2020)

^e^Ohm et al. (2015)

Mukhtar et al. (2019) said that the quality of solid biomass fuels is determined by the contents of their moisture (MC) and fixed carbon (FC). Moreover, when high moisture is associated with low fixed carbon, there is high likelihood to obtain low heating value. High moisture also induces biological activity during storage with the consequence of the loss of energy through evaporation during combustion which limits the process efficiency.

### Elemental constituents

The elemental constituents help to predict the properties of a fuel. The major elements in a fuel are carbon, oxygen, hydrogen, sulphur, and nitrogen and these are usually determined with the elemental analyser. The analysis involves the studies of a fuel measured at specified conditions according to EN 15104: 2011 and ISO 16948 (2015). the determination of carbon, hydrogen and oxygen is important for quality control and the results can be used as input parameters for calculations applied to the combustion and pollution potential of solid biofuels. The carbon content is required for the determination of CO_2_ emissions. Hydrogen content is also important for the calculation of the net heating value while the environmental importance of the nitrogen content was linked to the formation and emissions of NO_X_ (Demirbas, 2007). Previous studies have shown that biomass has high value of carbon, hydrogen, and oxygen and low nitrogen and sulphur values (Nunes et al. 2021). Table 7 presents the effect of torrefaction on carbon, hydrogen, oxygen, sulphur, and nitrogen contents and heating values of some feedstocks.

The energy released per unit mass during combustion of carbon is due to the energy in C–C bonds in place of O–H, C–H, and C–O bonds (Mukhtar et al. 2019). The adjudgment of a solid fuel quality as good was characterized by low hydrogen to carbon and oxygen to carbon ratios which are maximized through torrefaction (Mukhtar et al. 2019). The amount of carbon and hydrogen contents in biomass influences its heating value directly while the contents of oxygen and nitrogen have an inverse relationship with the heating value. Also, the high content of oxygen in raw feedstock reduces the number of C–H bonds, heating value, energy density, and thermal stability (Demirbas, 2007).

### Strength properties

Density, durability index, compressive strength and water resistance index are some of the important physico-mechanical properties that are studied to assess the strength and compactness of high-quality briquettes. The increase in the torrefaction temperature of some briquettes from 225 to 275 °C resulted in the decrease in their compressive strength from 9.61 to 3.41 MPa (Fuad et al. 2020). So, the compressive strength of the torrefied briquettes was higher than that of the raw briquettes (Faizal et al. 2018). The increase in strength can, however, only be attained if the biomass feedstock was torrefied before densification. Moreover, in the work of Akogun et al. (2020), the durability and water resistance indices of briquettes produced from the blends of the torrefied cassava peel and sawdust wastes also increased with the increase in the torrefaction temperature. Generally, the value of the durability index of a fuel is a function of the value of the compressed and relaxed density. For better clarification, Bai et al. (2017) stated that the sprinkling of 10% deionized moisture on torrefied samples prior to densification improves the durability index and other physico-mechanical properties of briquettes produced. However, in the work of Fuad et al. (2020) the densities of the torrefied palm kernel shell briquettes slightly decreased in comparison to that of the raw briquettes from 846 to 753 kg/m^3^. This was likely due to the densification of the palm kernel shell into briquettes before they were subjected to torrefaction process.

### Colour transformation and energy density

The colour of torrefied briquettes is visually transformed from light to dark brown when there is an increase in the torrefaction temperature. The change is due to the increase in the carbon content when the fuel was subjected to torrefaction (Nyakuma et al. 2015). Furthermore, the densified fuel becomes darker at different carrier gas flow rates during. Fuad et al. (2020) reported that the mild level of darkness obtained for briquettes with the increased gas flow rate reveals that the fuel may not be fully carbonized leading to more unreactive atmosphere during torrefaction. Generally, torrefaction removes light volatiles present in hemicellulose and cellulose of biomass, resulting in products with higher energy density and heating value. The feedstocks with higher hemicellulose and cellulose contents will give lower solid yield and energy density compared with those with lower cellulose and hemicellulose contents (Shahbaz et al. 2020).

### Applications of torrefied briquettes

Torrefied briquettes are useful for domestic and industrial applications. This fuel is often produced and used as replacement of firewood and charcoal. Meanwhile, with the current tide in the price and shortage of fuel, people are searching for cheap, clean, and alternative fuels to fill this gap. The viable usage of torrefied briquettes is in their joint combustion with coal in coal thermal engines, gasifiers, and boilers (Ohm et al. 2015). The high fuel quality capacity of this fuel makes it acceptable for heating applications for electricity generation and industrial applications. Other applications of torrefied briquettes are in cooking, roasting, water heating, tea drying and other agricultural processing (Chen et al. 2015; 2021; Wild and Calderón, 2021).

### Findings, drawbacks, and way forward in biomass and briquette production

Briquetting technology has been largely unsuccessful in Africa. This is because most of the heating energy needs of rural people in Africa are met by the direct burning of wood and loose biomass using low-efficiency stoves (Obi et al., 2022). The limited production and use of briquettes could be linked to lack of developed supply chain structure. Ibitoye et al. (2021) also corroborated that success is recorded mostly in the developed nations on biomass densification. The developing nations experienced drawbacks due to poor management, inadequate equipment, and high investment costs. The findings, drawbacks, and way forward in biomass and briquette production are discussed in what follows:

1. Land inaccessibility for biomass cultivation for bioenergy production: Access to land ownership has created restrictions to commercial farming leading to unsustainable biomass production in many developing countries. These restrictions were associated with communal, tribal, or ethnic conflicts. Land tenure issues should be resolved to give room for land acquisition for biomass plantation.
2. High cost of biomass production for fuel: The cost of production through biomass cultivation or processing is on the high side in comparison with the cost implications and energy that will be generated. The relevant government agencies involved should assist in terms of subsidy.
3. High cost of infrastructure and machines: Biofuel production in many parts of Africa is highly challenging in terms of good road network, constant electricity and an easy to operate and cheap machinery for torrefaction and briquette production. Special financial support should be provided for the procurement of expensive equipment. The design of machines using locally available materials should be encouraged by the government or corporate organizations. Necessary infrastructure should also be provided including access to good transport network for easy transfer of goods and services.
4. Security challenge: It is no longer news that farmers in many developing countries are confronted by rampage on their farmland due to incessant attacks by killer herdsmen, bandits, and terrorists. They usually run away to protect their lives from untimely death and save themselves from stress involved in land cultivation. This has unfortunately reduced the cultivation of energy crops and the agricultural wastes or residues which would be eventually generated. Grazing lands in form of ranching should be provided for herders to feed their animals to prevent invasion of farmland by animals. Security operatives should be provided to prevent the activities of killer herders and bandits.
5. Government policy: The intervention of relevant government agencies as regards biomass utilization for clean and sustainable energy is not encouraging in many parts of the world. They have continued to promote the use of fossil resources as their primary source of energy despite the environmental impact and the impending destruction of the ozone layer it could cause. Government should create masses-friendly policies that will encourage and promote biofuel technologies. Moreover, tax exemption would also encourage the technocrats in the biomass energy industry.
6. Competition between biomass cultivation for food and fuel: Food is an essential aspect of living for man and livestock and man will prefer to grow crops for food rather than cultivate biomass for energy generation. The utilization of biomass for biofuel is usually seen as threat to food security. Government and energy related industries should therefore consider the massive cultivation and utilization of nonfood biomass such as jatropha, sunflower, etc., as feedstock.
7. Difference in the combusting properties of biomass and coal: Coal powered engines are only designed to use coal as fuel because raw biomass or briquette has some combusting properties such as high volatiles and moisture content which make its application challenging as feedstock. More research on combustion indices (Conag et al., 2017, 2018) and process optimization should be carried out on torrefied briquettes to minimize emissions and cost before they are used as feedstock in coal thermal plant.
8. Loss of binding agents: Biomass are known to contain lignin which is a major natural binding constituent of biomass. However, this natural binder is lost during torrefaction making the briquetting of torrefied products difficult. The use of organic or inorganic binders can help to enhance binding abilities during densification. Water preconditioning of torrefied products is also recommended (Bai et al., 2017).
9. Upscaling: Torrefaction and briquetting are majorly at research stage in developing countries and many parts of the world. Upscaling the torrefaction and briquetting conversion facilities from research stage into industry ready product is recommended.

## Conclusion

Significant research efforts have been made on torrefaction and briquetting technologies towards sustainable energy development for heat and power generation. This paper has provided an overview on torrefied briquettes from agricultural wastes. The review discussed lignocellulosic biomass composition and challenges, torrefaction pre-treatment, briquetting of torrefied products, the parameters influencing the quality of torrefied briquettes, behaviour of briquettes, applications of torrefied briquettes and way forward in the briquetting sector. The pre-treatment of several agricultural wastes for useable fuel by torrefaction and densification, improved the performance of the produced briquette thus, providing a viable opportunity for agricultural waste utilization that would fulfil the requirements for commercialization in line with standard procedures. Few of the important quality parameter affected by torrefaction X-rayed in this study include compositional constituents, combustion indices, colour change, energy density, carrier gas, flow rate, heating rate, heating value, strength properties, proximate characteristics, elemental distribution, and the degradation of briquette constituents. The wide gap between briquette production and its adoption in developing countries should be bridged through government intervention programmes and policies. The masses should also be motivated to upwardly drive the clean-energy industry. Meanwhile, people centred and favourable policies in the form of financial support, awareness creation, technology transfer, and improved power supply can significantly improve the development of rural briquette industry in developing countries. The necessary steps to upscaling the torrefaction and briquetting bioconversion process from research stage into industry ready product should be followed accordingly.

## Data Availability

Not applicable. It is a review paper and data were obtained from published papers.
